# Generation and Staging of Human Retinal Organoids Based on Self-Formed Ectodermal Autonomous Multi-Zone System

**DOI:** 10.3389/fcell.2021.732382

**Published:** 2021-09-22

**Authors:** Jinyan Li, Yijia Chen, Shuai Ouyang, Jingyu Ma, Hui Sun, Lixia Luo, Shuyi Chen, Yizhi Liu

**Affiliations:** State Key Laboratory of Ophthalmology, Zhongshan Ophthalmic Center, Sun Yat-sen University, Guangzhou, China

**Keywords:** human retinal organoid, RPE, ciliary margin, photoreceptor cell, SEAM system, retinogenesis

## Abstract

Methods for stem cell-derived, three-dimensional retinal organoids induction have been established and shown great potential for retinal development modeling and drug screening. Herein, we reported an exogenous-factors-free and robust method to generate retinal organoids based on “self-formed ectodermal autonomous multi-zone” (SEAM) system, a two-dimensional induction scheme that can synchronously generate multiple ocular cell lineages. Characterized by distinct morphological changes, the differentiation of the obtained retinal organoids could be staged into the early and late differentiation phases. During the early differentiation stage, retinal ganglion cells, cone photoreceptor cells (PRs), amacrine cells, and horizontal cells developed; whereas rod PRs, bipolar cells, and Müller glial cells were generated in the late differentiation phase, resembling early-phase and late-phase retinogenesis *in vivo*. Additionally, we modified the maintenance strategy for the retinal organoids and successfully promoted their long-term survival. Using 3D immunofluorescence image reconstruction and transmission electron microscopy, the substantial mature PRs with outer segment, inner segment and ribbon synapse were demonstrated. Besides, the retinal pigment epithelium (RPE) was induced with distinct boundary and the formation of ciliary margin was observed by co-suspending retina organoids with the zone containing RPE. The obtained RPE could be expanded and displayed similar marker expression, ultrastructural feature and functional phagocytosis to native RPE. Thus, this research described a simple and robust system which enabled generation of retina organoids with substantial mature PRs, RPE and the ciliary margin without the need of exogenous factors, providing a new platform for research of retinogenesis and retinal translational application.

## Introduction

The human eye is composed of the refractive system (lens and cornea) and the visual neural system (retina). Being the sensory structure in the eye, the retina is highly stratified and retinogenesis is orchestrated by a series of networks of extrinsic and intrinsic signaling. During retinogenesis, the eye field first appears as an optic vesicle and eventually invaginates into a double-layered optic cup. The outer layer gives rise to the retinal pigment epithelium (RPE) and the inner layer becomes the neural retina (NR), which further form the lamellae that comprise the six retinal cell types (Chow and Lang, 2001).

Substantial progress has been made in the modeling of three-dimensional retinal organoids “in a dish.” Up to now, existed retinal organoid induction strategies could be briefly divided into four categories, including direct 3D induction-based, embryoid body-based, lumen cyst-based and confluent adherent culture-based induction system (Meyer et al., 2009, 2011; Nakano et al., 2012; Reichman et al., 2014; Zhong et al., 2014; Mellough et al., 2015; Lowe et al., 2016; Sridhar et al., 2016; Gonzalez-Cordero et al., 2017; Wahlin et al., 2017; Hallam et al., 2018; Ovando-Roche et al., 2018; Phillips et al., 2018; Capowski et al., 2019; Shrestha et al., 2019; Pan et al., 2020; Regent et al., 2020; Zhang and Jin, 2021); these can mimic retinogenesis *in vitro* and have malignant application value (see the systematic reviews (Artero Castro et al., 2019; Jin et al., 2019; Kruczek and Swaroop, 2020; Nguyen et al., 2020; O’Hara-Wright and Gonzalez-Cordero, 2020; Sharma et al., 2020). To be noted that most of these existed protocols required various exogenous factors treatment, including growth factors and small-molecule inhibitors, which might render undesired complexity when applied for candidate signaling molecule research or drug screening (Hyun, 2010; Daley, 2012; Wan et al., 2015). Thus, we aimed to set up an exogenous-factors-free retina organoids construction system.

Nishida’s research group reported an interesting two-dimensional induction system, termed “self-formed ectodermal autonomous multi-zones” (SEAMs) (Hayashi et al., 2016, 2017), which was capable of generating substantial colonies comprising multiple ocular lineages without the need of exogenous factors. The generated corneal components have the capacity to recover corneal function, indicating its potential for clinical translation of the SEAM system. Hence, we wondered whether substantial and mature retinal organoids could be also achieved by modifying the SEAM system.

In this study, we established a robust method for retinal organoids induction based on the SEAM system. After our modifications, three-dimensional retinal organoids were substantially induced without the need of exogenous factors. Moreover, we showed that the morphogenesis of the obtained organoids could be staged into two phases, resembling to early-phase and late-phase retinogenesis *in vivo*, and developed substantial mature PRs, spontaneous differentiated RPE as well as the ciliary margin like structure. Taken together, we herein reported an exogenous-factors-free and robust method for the induction of retinal organoids, which were additional to the field of retina organoid induction system and might be a powerful tool for retinogenesis modeling and drug screening.

## Materials and Methods

### Human Embryonic Stem Cell Culture

The hESC H9 cell line was a generous gift from Xiaoyan Ding at the Institute of Biochemistry and Cell Biology, obtained from the National Stem Cell Bank (c/o WiCell Research Institute). The cells were cultured on Matrigel (BD Biosciences)-coated plates in mTeSR medium (STEMCELL Technologies). Cells were regularly passaged and approximately 4.5–6.0 × 10^4^ cells per well were cultured in 6-well plates to promote colony formation.

### Retinal Organoid Differentiation

The starting cell density was crucial, as a cell density either below 70% or above 90% affected the differentiation efficiency. hESCs were transferred to SEAM medium containing G-MEM (Gibco), 10% knockout serum replacement (KSR; Life Technologies), 0.1 mM non-essential amino acids (NEAA; Life Technologies), 1 mM sodium pyruvate (Life Technologies), 1% penicillin-streptomycin solution (PS; Life Technologies), and 55 μM 2-mercaptoethanol (Life Technologies) for the first Week 2–3 to form cell clusters. After colony formation, the induction medium was switched to retinal induction medium (RDM) containing DMEM: F12 3:1, 2% B-27 supplement (Thermo Fisher Scientific), 0.1 mM NEAA, and 1% PS. Around Week 4–6, the cup-like retina aggregates became visible and were isolated using fine forceps (Dumont) under a dissecting microscope. We modified the long-term suspension culture strategy by using low-attachment 96-well plates in RDM supplemented with 10% fetal bovine serum (FBS, HyClone), 100 mM taurine (Sigma) and 0.1 mM GlutaMAX (Life Technologies) (retinal maturation medium (RMM). All-*trans* retinoic acid (RA, 1 μM, Sigma) was additionally added after 2 weeks of suspension culture. An Axio Observer D1 microscope (Carl Zeiss) was used to acquire phase-contrast and bright-field images.

### Retinal Pigment Epithelium Expansion

The RPE spheres were manually collected from the retinal organoids at Week 5–7. 10–20 spheres were digested with Accutase (Innovative Cell Technologies) at 37°C for 8 min into individualized cells and seeded into a Matrigel pre-coated well of a six-well plate. During the first week, RDM supplemented with 10% FBS and 100 mM taurine was used. When the cells reached to 100% confluence, FBS was removed from the medium, which was beneficial for RPE maturation. The medium was exchanged every 2–3 days.

### Immunofluorescence Staining

Immunofluorescence staining of cells cultured on plates was performed as previously described (Han et al., 2018). In brief, cells were fixed in 4% paraformaldehyde (PFA; Sigma) at room temperature for 10 min and permeabilized with 0.5% Triton X-100 (Sigma) for 10 min. Cells were then blocked by 5% normal donkey serum (NDS; Jackson ImmunoResearch) for 30 min. Subsequently, the cells were incubated with primary antibodies overnight at 4°C, followed by incubation with the corresponding Alexa Fluor-conjugated secondary antibodies at room temperature for 1 h. The nuclei were counterstained with DAPI (Invitrogen). Fluorescence images were acquired using a ZEISS Axio Observer Z1 (Carl Zeiss).

The obtained retinal organoids were fixed in 4% PFA at 4°C overnight, and immersed in 15% sucrose followed by 30% sucrose in PBS before cryopreservation. The frozen sections were incubated in citrate buffer (pH 6.0) at 95°C for 30 min and cooled to room temperature for antigen retrieval. The sections were then incubated with primary antibody diluted in 5% NDS at 4°C overnight. After washing with PBS-Tween, the sections then were incubated with Alexa Fluor-conjugated secondary antibodies for 1 h. The nuclei were counterstained with DAPI. Fluorescence images were acquired with ZEISS LSM880 confocal microscope (Carl Zeiss).

For whole-mount immunocytochemistry, the organoids were fixed in 4% PFA at room temperature for 10 min. After washing with PBS containing 1% Triton X-100 (PBSTr), the organoids were rinsed with H_2_O and treated with acetone at –20°C for 7 min. The organoids were incubated with the primary antibody diluted in PBSTr containing 10% NDS overnight at 4°C. On the following day, the organoids were washed with PBSTr (three times for 30 min). Incubation with the secondary antibody was performed at room temperature for 3 h. The nuclei were counterstained with DAPI. A ZEISS LSM880 confocal microscope were used to acquired fluorescence images using the Z-stack scan modes, and 3D reconstruction was performed by Zen software (Carl Zeiss). Alexa Fluor 488 or 568-conjugated donkey anti-rabbit, mouse, sheep or goat secondary antibodies (1:500; Invitrogen) were used. The primary antibodies, suppliers, and dilutions used are presented in Supplementary Table 1.

### Transmission Electron Microscopy

Samples were fixed in EM fixative (2.5% glutaraldehyde/2% PFA) at 4°C and immediately sent for dehydration, embedding, sectioning and staining at Electron Microscopy Core Facility of Sun Yat-sen College of Medical Science, Sun Yat-sen University (Guangzhou, China). Ultrastructural analysis were performed by transmission electron microscope (Tecnai G2 Spirit; FEI, Inc., Carlsbad, CA, United States).

### Phagocytosis Assay

Phagocytosis Assay was performed using 1 μm polystyrene FluoSpheres (Invitrogen) (Shrestha et al., 2020). In brief, confluent monolayers of RPE cells were incubated with FluoSpheres (cell counts/particles, around 1:100) at 37°C for 4 h. Then, the cultures were washed, fixed with 4% PFA and performed immunofluorescence staining as described above. Fluorescence images were acquired with ZEISS LSM880 confocal microscope.

### Statistical Analysis

Statistical comparisons were conducted using GraphPad Prism (GraphPad software). All experiments were conducted at least in triplicate. The values are expressed as mean ± SEM (standard error of mean) or mean ± SD (standard deviation). *P* < 0.05 was considered statistically significant.

## Results

### Robust Retinal Organoid Induction After Self-Formed Ectodermal Autonomous Multi-Zone System Modification

**FIGURE 1:**
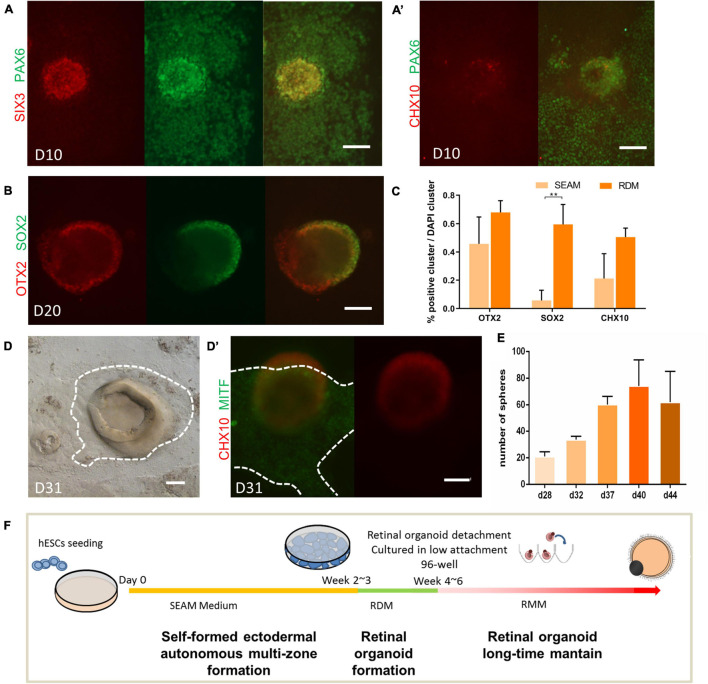
Robust retinal organoids induced by the modified SEAM method. **(A,B)** Expression pattern of key transcription factors during the induction of retinal organoids. Immunofluorescent staining of eye field marker SIX3 (red) and PAX6 (green) on Day 10 **(A)**; the retinal progenitor cell marker CHX10 (red) on Day 10 **(A’)**; the neural progenitor cell markers OTX2 (red) and SOX2 (green) on Day 20 **(B)**. Scale bar = 100 μm. **(C)** The ratio of OTX2-, SOX2-, and CHX10-labeled clusters to total clusters (marked by DAPI) on Day 28 in the SEAM- and RDM-treated groups, respectively. The bars represent the mean ± SEM. **P < 0.01 (*n* = 3). **(D)** Bright-field imaging showing the derivation of cup-like neural structure and the surrounding pigment cell on Day 31. The identification of the retinal progenitor cell marker CHX10 (red, in the 3D cup-like neural structure) and retinal pigment epithelial cell marker MITF (green, at the bottom of the 3D cup-like neural structure) **(D’)**. Scale bar = 100 μm. **(E)** Average number of retinal organoids obtained per well in 6-well plates on Day 28, Day 32, Day 37, Day 40, and Day 44 (*n* = 3). **(F)** Schematic diagram of the induction of human retinal using the modified SEAM method.

First, to examine how well the retinal fate was established in the SEAM system, we examined the expression of retina development-related transcription factors in the SEAM system. As shown in Figure 1A, after approximately 2 weeks into the SEAM induction, cell aggregates had acquired eye field fate, expressing PAX6 and centrally located SIX3 (Bailey et al., 2004; Miesfeld and Brown, 2019). A few Chx10^+^ cells (a specific markers of retinal progenitor cells) were observed at this time (Figure 1A’), indicating the low efficiency of retinal fate induction. Hence, we wondered whether we could enhance the retinal fate induction by replacing the original SEAM medium with retinal differentiation medium (RDM), which is known to induce retinal progenitor cells (Zhong et al., 2014). As shown in Figure 1B, after RDM replacement, aggregates expressing neural progenitor marker SOX2 and OTX2 robustly emerged and gradually developed a horseshoe/dome-shaped structure. Compared with the original SEAM protocol, the use of RDM improved the number of clusters expressing OTX2, SOX2, and CHX10, which are crucial for retinal progenitor cell differentiation (Figure 1C; Livne-Bar et al., 2006; Danno et al., 2008).

After 3–4 weeks of differentiation, distinct colonies with multiple-zones had started to form. Cup-like structures were observed in the center of these colonies. The paracentrally distributed pigmentation were seen as well, surrounded by an identifiable border (Figure 1D). Immunofluorescence showed that the three-dimensional structure was CHX10-positive and the adjacent pigmented zone was MITF-positive, indicating the cell fate specification of NR and RPE, respectively (Figure 1D’; Bharti et al., 2008; Zou and Levine, 2012). Under RDM treatment, new retina organoids of various sizes emerged continuously from around week 4–6. After 40 days of differentiation, approximately 74 colonies with multiple-zones (±19, *n* = 4 technical replicates per well, in six-well plates) were harvested (Figure 1E). Thus, as illustrated in Figure 1F, without the need of exogenous factors, we achieved a substantial number of retinal organoids with RPE by modification of the SEAM protocol.

### Staging of Retinal Organoid Morphogenesis

The cornea precursors cells in the SEAM system have been demonstrated their full potential to generate a mature corneal epithelium (Hayashi et al., 2016), but the retinal components have not. Thus, we next sought to investigate the differentiation capacity of the retinal organoids derived from the modified SEAM protocol. To normalize for the inconsistency of the retinal organoid formation, we defined the day of isolation of the suspension culture as Day 0.

**FIGURE 2:**
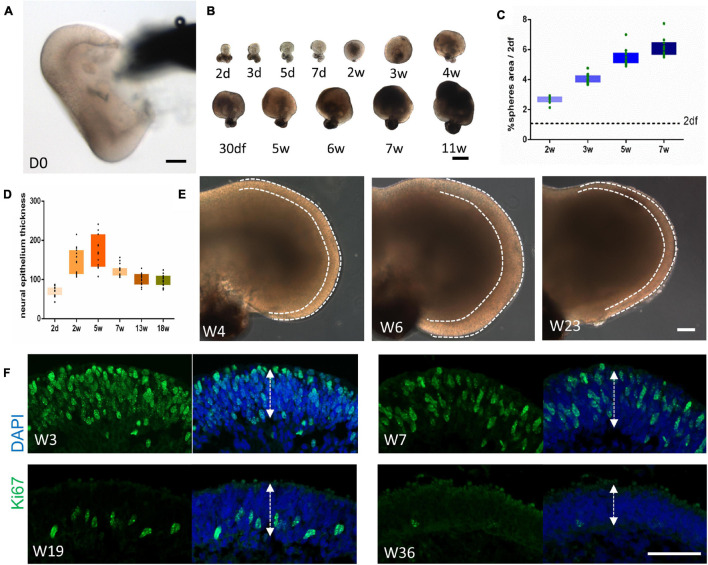
Morphogenesis of retinal organoids. **(A)** Bright-field imaging showing the cup-like structure of the retinal organoid immediately after isolation. Scale bar = 100 μm. **(B)** Variation in the growth rate of retinal organoids at different time points. Scale bar = 500 μm. **(C,D)** The growth rate was quantified by sphere size [**(C)**, *n* = 10 retinal organoids from three independent experiments] and neural retina thickness [**(D)**, *n* = 15 retinal organoids from three independent experiments]. **(E)** Representative images at Week 4, Week 6, and Week 23 showing the changes in the thickness of the neural retina (dashed line). Scale bar = 100 μm. **(F)** Ki67 immunostaining of the neural retina at Week 3, Week 7, Week 19, and Week 36, indicating the downregulation of proliferation as differentiation proceeded. The neural retina thickness was measured as illustrated by the arrows. Scale bar = 50 μm.

The morphogenesis of retinal organoids was then documented. All organoids possessed a cup-like or horseshoe-like structure right after pinching (Figure 2A). As shown in Figure 2B, the organoids increased in size in the first 7–11 weeks, but with different growth rates. The growth rate was quantified by sphere size and neuroepithelium thickness. During the first 5–6 weeks, the organoids grew rapidly, with relatively slower growth afterward (Figure 2C). A similar increase was found for the thickness over the first 5 weeks; subsequently, it started to thin (Figure 2D). Moreover, the lamination of the neuroepithelium was well-defined during the rapid growth period (before Week 6), and generally became blurred (Figure 2E). Along with the morphologic changes, immunostaining for Ki67 showed the extensive proliferative activity throughout the neuroepithelium in Week 3–7. As the maintenance time was prolonged, Ki67^+^-cells could only be detected in the inner layer (Week 19) and were finally barely visible (Figure 2F), indicating the decreased proliferation of retinal cells. Hence, we defined two patterns of retinal organoids differentiation as early (Week 0–6/7) and late stage (after Week 6/7), respectively.

### Early-Stage Differentiation Resembled Early-Phase Retinogenesis *in vivo*

**FIGURE 3:**
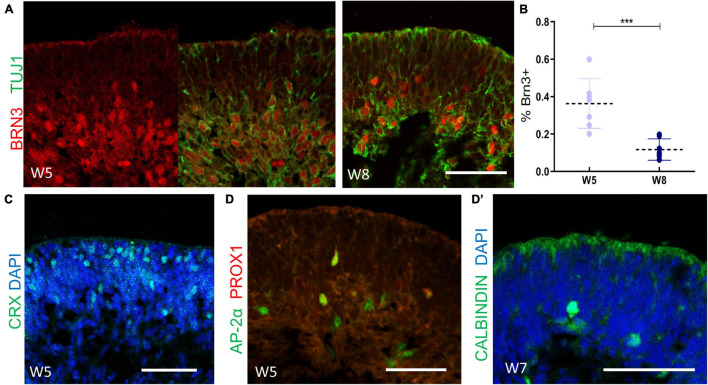
The cellular composition of retinal organoids during the early-stage differentiation. **(A)** The cellular dynamics of retinal ganglion cells were marked at Week 5 (BRN3, red; TUJ1, green), and Week 8 (BRN3, red; TUJ1, green). **(B)** Statistics analysis of the ratio of BRN3 + cells versus total cells (marked by DAPI) at Week 5 and Week 8, respectively. Mean ± SD, ***P < 0.0005. **(C)** Localization of CRX^+^ cone photoreceptor cells at Week 5. **(D)** The onset of AP2α + amacrine cells and AP2α^+^/PROX1^+^ horizontal cells were detected (AP-2α, green; PROX1-red) at Week 5 and confirmed by CALBINDIN (green, **D’**) staining at Week 7. Scale bar = 50 μm.

Retinal cell differentiation follows an established pattern *in vivo*. Retinal ganglion cells (RGCs), cone photoreceptor cells (PRs), horizontal cells, and amacrine cells originate from early retina progenitors in sequence (Jin and Xiang, 2017; O’Hara-Wright and Gonzalez-Cordero, 2020). In our system, BRN3^+^/TUJ1^+^ RGCs occurred first and were observed at 2 weeks after isolation. Subsequently, the number of BRN3^+^ RGCs increased sharply, lining the innermost layer of neuroepithelium. However, we noticed that the number of RGCs decreased (Figure 3A). As shown in Figure 3B, compared to that of Week 5, the average ratio of BRN3 + cells versus total cells (marked by DAPI) of Week 8 was significantly decrease (36.32% ± 13.22% at Week 5 and 11.72% ± 5.7% at Week 8, respectively, *n* = 7 independent replicates).

CRX^+^ cone PRs emerged after robust RGCs generation, starting from the basal side of the neuroepithelium and gradually migrating to the corresponding apical layers (Figure 3C). Furthermore, a small proportion of AP2α^+^ amacrine cells and AP2α^+^/PROX1^+^ horizontal cells (Zhong et al., 2014) were detected at Week 5, as further confirmed by CALBINDIN labeling (Figures 3D,D’). Therefore, these findings suggested that in early differentiation stage, RGCs, cone PRs, amacrine cells, and horizontal cells had developed, thus mimicking the early-born retinal lineage differentiation pattern during vertebrate retinogenesis.

### Late-Stage Differentiation Recapitulated Late-Phase Retinogenesis *in vivo*

**FIGURE 4:**
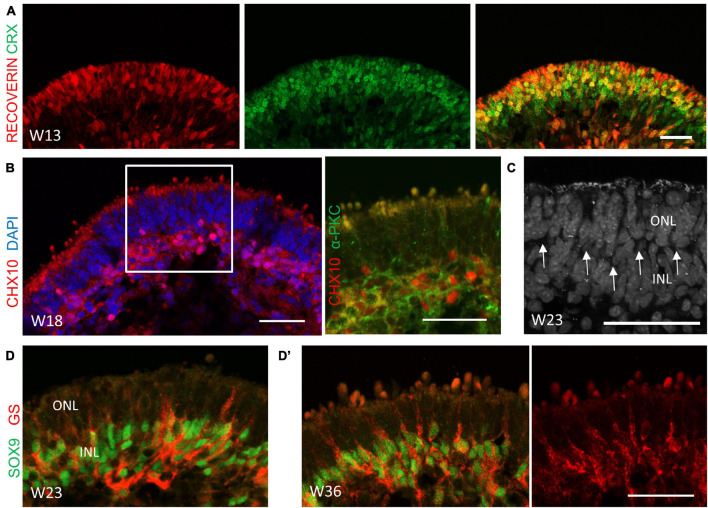
The cellular composition of retinal organoids during the late-stage differentiation. **(A)** Robust derived cone and rod photoreceptor cells at Week 13 as indicated by CRX (green) and RECOVERIN (red), respectively. Scale bar = 100 μm. **(B)** The co-localization of CHX10 (red) and α-PKC (green) in the presumptive inner nuclear layer (INL), suggesting the generation of bipolar cells at Week 18. **(C)** The nuclei-free layer between the outer nuclear layer (ONL) and the INL was illustrated by DAPI immunostaining, indicating the outer plexiform layer (OPL, arrowhead). **(D)** Müller glial cells were detected at Week 23 and were rapidly generated at Week 36 **(D’)**, as shown by immunostaining of the Müller glial cell markers SOX9 (green) and GS (red). Scale bar = 50 μm.

We continued to study the cellular dynamics of retinogenesis in the late-stage differentiation of our system. In addition to CRX^+^ cells, a wave of Recoverin^+^ cells were observed at the apical side at Week 13 (Figure 4A), implying the generation of rod PRs. CHX10 and SOX9 are markers of retinal progenitor cells during development, and their expression are restricted to bipolar cells and Müller glial cells in the mature retina, respectively (Liu et al., 1994; Poche et al., 2008). In our system, CHX10- or SOX9-labeled retinal progenitor cells were found throughout the neuroepithelium until Week 5 (Supplementary Figure 1A). Their expression were gradually restricted in the deep, relative to the outermost layer during the late differentiation stage (Supplementary Figure 1B). CHX10^+^/α-PKC^+^ bipolar cells were generated at Week 18 and migrated to a progressively distinguishable layer, which was indicative of the developing presumptive inner nuclear layer (INL) (Figure 4B). In addition, a thin, nuclei-free layer was gradually established, in a position indicative of the outer plexiform layer (Figure 4C; Capowski et al., 2019). A few SOX9^+^/GS^+^ Müller glia cells could be detected at Week 23 (Figure 4D). Subsequently, more Müller glia cells were generated, confined to a near single layer of cells within the presumptive INL, with their projection extending to the outermost layer in a radial orientation (Figure 4D’). Collectively, these results suggested that during the late-stage differentiation, rod PRs, bipolar cells, and Müller glia cells were generated, with the development of the INL and the outer plexiform layer, which corresponded to late-born retina differentiation phase *in vivo*.

### Efficient Long-Term Survival and Maturation of Photoreceptor Cells

To make retinal organoids capable of long-term survival, the maintenance protocol was further modified. As spheres in a mixed suspension culture tend to adhere to each other, resulting in disruption of the laminar morphology, we transferred them into low-attachment 96-well plates after isolation to provide an undisturbed environment. It turned out this small change efficiently promoted survival, as well as reducing reagent costs.

**FIGURE 5:**
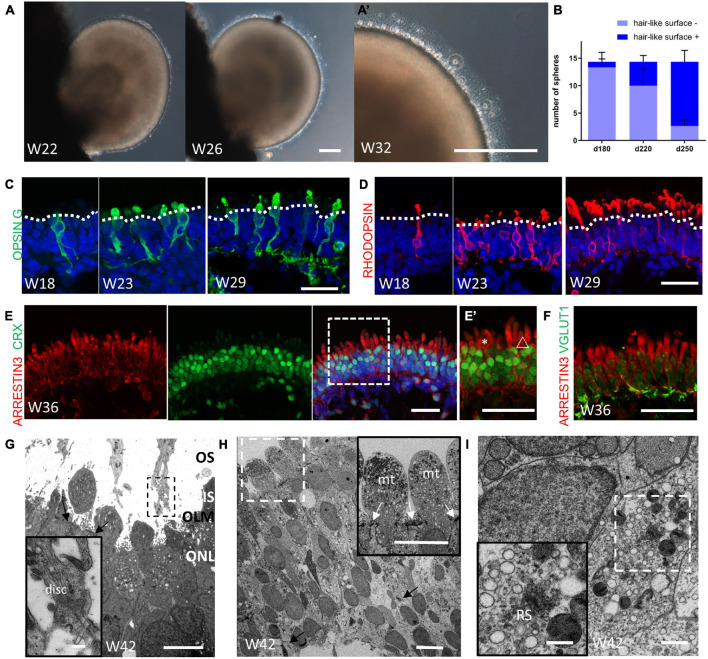
Maturation of photoreceptor cells in retinal organoids. **(A)** Representative images showing the onset of hair-like microvilli on the surface of the retinal organoid at Week 22, Week 26, and **(A’)** Week 32. Scale bar = 100 μm. **(B)** Progressive increase in the number of retinal organoids with hair-like microvilli (*n* = 3 independent experiments per group; each group contained 18, 12, and 13 retinal organoids, respectively). **(C,D)** Representative images showing the maturation of cone and rod PRs, as indicated by the mature PRs markers Opsin Green/Red (**C**, Opsin G/R, green) and RHODOPSIN (**D**, red) at Week 18, Week 23, and Week 29. Scale bar = 50 μm. **(E)** Immunofluorescent staining of the PR marker ARRESTIN 3 (red) and CRX (green) showed that a 4/5-nuclei–thick layer was formed. **(E’)** Higher magnification of **(E)** showing the distinct morphology of the cone outer segment (asterisks) and the rod outer segment (triangle). Scale bar = 50 μm. **(F)** The co-staining of ARRESTIN 3 (red) and vesicular transporter marker VGLUT1 (green) indicated the formation of the PR synapse. Scale bar = 50 μm. **(G)** Electron microscopy showed the formation of outer segments-like (OS) and inner segments-like structure (IS), outer limiting membrane (OLM, indicated by black arrow) and outer nuclear layer (ONL). Scale bar = 5 μm. The insert image showed the infolding disk-like structure in OS. Scale bar = 0.5 μm. **(H)** The 4/5-nuclei–thick ONL was shown. The black arrow indicated that the ribbon synapses-like structure were present at basal side of photoreceptors. Scale bar = 10 μm. The insert image showed the mitochondria-rich inner segment-like structure. The white arrow indicated the OLM. Scale bar = 2 μm. **(I)** The clearer image of ribbon synapse-like structure (RS) was shown, surrounded by various vesicles. Scale bar = 500 nm (The insert image scale bar = 50 nm).

At Week 21 (or earlier in some cases), the organoids began to grow hair-like microvilli on the surface, which has been shown to represent the developing PR outer segments (Figures 5A,A’; Gonzalez-Cordero et al., 2017; Phillips et al., 2018; Capowski et al., 2019). Most of the organoids were able to develop these hair-like surface appendages in the separated culture (Figure 5B). Indeed, expression of the cone PR marker Green/Red Opsin and the rod PR marker Rhodopsin clearly showed the typical morphogenesis of both types of PRs (Figures 5C,D). CRX^+^/Arrestin 3^+^ PRs were arranged in a continuous, uniform, 4/5-nuclei–thick layer when the outer segments formed (Figure 5E,E’). The vesicular transporter marker VGLUT1 was shown to be lining underneath Arrestin 3^+^ cells, indicating that the PR synapse had been generated (Figure 5F). Transmission electron microscopy analysis showed that the hair-like microvilli, which extended through the outer limiting membrane, was found to contain mitochondria-rich inner segments-like and disk-containing rudimentary outer segments-like structures, indicating the maturation of photoreceptors (Figures 5G, H). Besides, ribbon synapse, a specialized form of synapse connecting photoreceptor cells, bipolar and the interneurons, was found at the basal side of photoreceptors (Figures 5H, I; Capowski et al., 2019). Taken together, these results have demonstrated that the obtained retinal organoids were capable to develop photoreceptor cells with a high degree of maturation containing outer segment, inner segment and the synaptic connectivity between PR axon terminals and cells of the INL.

**FIGURE 6:**
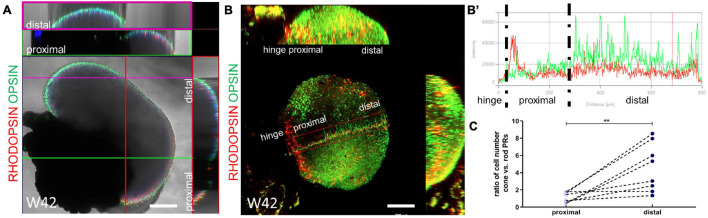
The uneven distribution of rod and cone photoreceptor cells. **(A)** The ortho X-Z display of a random section of the organoid at Week 42 showed that the hinge distal part was mainly composed of cone PRs (Opsin B/G/R, green, purple frame) and rod PRs (Rhodopsin, red, green frame) mainly located at the hinge proximal part. **(B)** Ortho display of maximum intensity projection of the whole organoid at Week 42. The fluorescence intensity from the hinge proximal to distal part (marker by the red box) was calculated in **(B’)**. Scale bar = 200 μm. **(C)** The ratio of the numbers of cone PRs versus rod PRs were paired calculated in the proximal and distal part, respectively. **P < 0.005.

To have a comprehensive analysis of the distribution of rod and cone PRs in our retinal organoids, we performed whole-mount immunostaining using mature markers of both types of PRs. The 3D reconstruction showed the high performance of our retinal organoid induction protocol, as the external surface of our organoids was densely filled with needle-like mature rod and cone PRs. Furthermore, rod and cone PRs were unevenly distributed in our system (Figures 6A,B and Supplementary Figure 2). The fluorescence intensity assay showed that the rod PRs were mainly located in the RPE proximal part, whereas the cone PRs were in the distal part (Figure 6B’). The average ratio of the numbers of cone PRs versus rod PRs were 1.12 (±0.60, ranging 0.44–1.80, *n* = 8) in the proximal part and 4.56 (±2.29, ranging 1.34–8.54, *n* = 8) in the distal part, respectively (Figure 6C). Taken together, these results indicated the potential of long-term survival and the maturation of the PRs obtained in our system.

### Autonomous Generation of Retinal Pigment Epithelium and the Ciliary Margin

**FIGURE 7:**
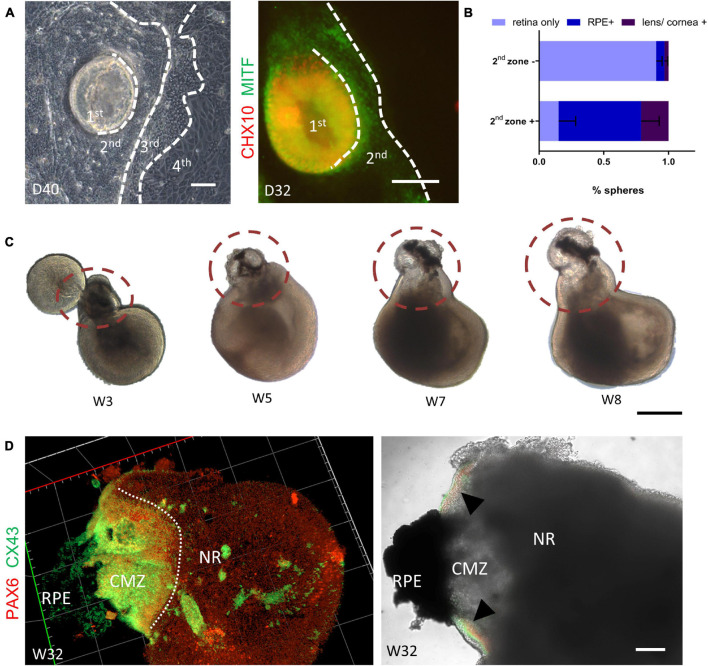
Autonomous generation of the retinal pigment epithelium and the ciliary margin. **(A)** Representative images showed the autonomous formation of a four concentric-zone cluster. Immunofluorescent staining showed that zone 1 and zone 2 were CHX10- (green) and MITF- (red) positive, respectively. Scale bar = 100 μm. **(B)** Compared with isolation without zone 2 [zone 2 (–)], isolation of retinal organoids with zone 2 [zone 2 (+)] generated more RPE spheres (*n* = 3 independent experiments per group; each group contained 20 or more retinal organoids). **(C)** Representative images showing the gradual formation of the peripheral neural retina between the neural retina and the RPE (dashed circle). Scale bar = 500 μm. **(D)** Co-staining of the ciliary margin marker PAX6 (green) and CX43 (red) confirmed the generation of ciliary margin zone at Week 32 (triangle). Scale bar = 200 μm.

As the original SEAM system was able to generate multiple ocular cell lineages, we wondered whether we could produce other ocular cell lineages after modification. As shown in Figure 7A, when the centrally located retinal spheres were developing, the peripheral domain progressively formed three other identifiable concentric zones within 4–6 weeks. Unlike the original SEAM methods, cells in zone 1 were Chx10^+^ NR progenitor cells and MITF^+^ RPE-commitment cells were in zone 2, with a distinct boundary to other zones. Apart from that, the distribution of PAX6^+^/p63^+^ corneal precursors in zone 3 and αA-crystallin^+^/PAX6^+^ lens primordial cells at the margin of zones 2 and 3 was similar to that of the original SEAM system (Supplementary Figure 3A; Shibata et al., 2018).

Therefore, by simply scratching the border around the underlying RPE domain (zone 2), it was simple to obtain retinal organoids suspended with the RPE-commitment cells. As illustrated in Figure 7B, isolation of zone 1–3 yielded retinal organoids with a 64% frequency (±14%, *n* = 3) of RPE spheres on Day 78, which was significantly higher than that without isolation of zone 2. Moreover, 21% (±6%, *n* = 3) of lens/corneal spheres could be harvested (Figure 7B and Supplementary Figure 3B). Hence, these results demonstrated that the simultaneous differentiation of retinal organoids and RPE was achieved in our system.

Given that the interaction of NR and RPE during retinogenesis is crucial for the development of the ciliary margin (CM) *in vivo* (Cicero et al., 2009; Fischer et al., 2013), we examined the morphological dynamics of retinal organoids coupled with RPE spheres. During the first few days after zone 1–3 suspension, these semi-spherical aggregates gradually became spherical (Supplementary Figure 3C). As the suspension culture proceeded, part of the NR that was adjacent to the presumptive RPE region, extended, grew increasingly thinner, and finally obtained peripheral-NR-like morphology (Figure 7C), consistent with the researches of CM induction (Kuwahara et al., 2015; Kinoshita et al., 2016). Finally, a distinct extended domain expressing PAX6^+^/CX43^+^ (markers of the CM) were clearly observed at the junction between the NR and the RPE, indicating the formation of the CM-like structure in the retinal organoids (Figure 7D). Collectively, these results indicated that our retina organoid induction system enabled the simultaneous generation of RPE and the CM.

### Expansion and Validation of Retinal Pigment Epithelium Differentiation Capacity

**FIGURE 8:**
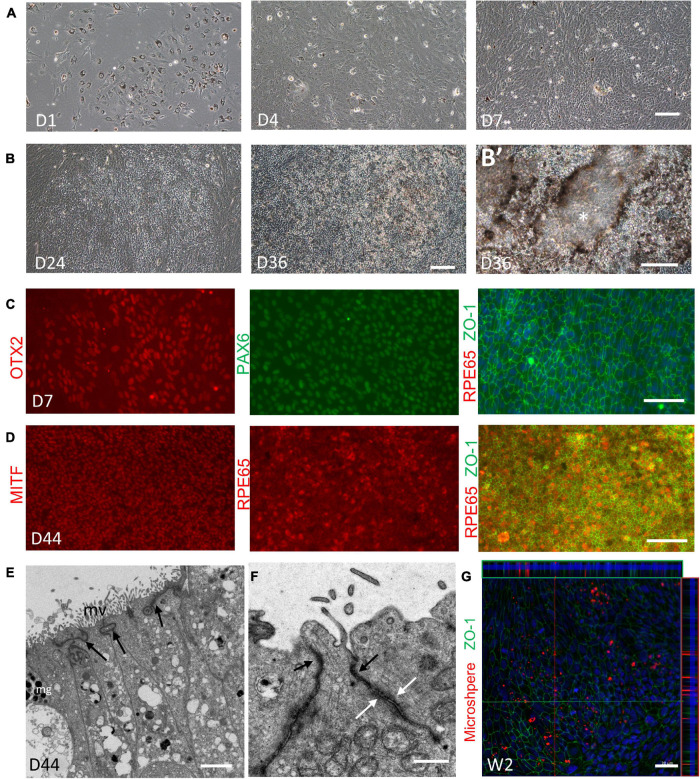
Expansion and validation of the retina pigment epithelium (RPE). **(A,B)** Representative images showing the expansion and pigmentation loss of the RPE on Day 1, Day 4, and Day 7 **(A)**; and the re-pigmentation on Day 24 and Day 36 **(B)**. **(B’)** Higher magnification images showing the formation of elevated domes (asterisks). **(C)** Expression of the naïve RPE marker OTX2 (red, left), PAX6 (green, middle) and the RPE marker ZO-1 (green, right) was shown on Day 7, while the mature RPE marker RPE65 (red, right) was not found. **(D)** On Day 44, RPE65 (red, left) coupled with the RPE marker MITF (red, middle) and ZO-1 (green, right) indicated the maturation of the RPE. Scale bar = 100 μm. **(E)** Electron microscopy showed that on Day 44, RPE sheet has developed melanin granules (mg), microvilli (mv) and the abundant tight junctions at the apical side (indicated by black arrow). Scale bar = 2 μm. **(F)** Different cell junctions of RPE were clearly shown (apical tight junctions, indicated by black arrow; desmosomes, indicated by white arrow). Scale bar = 500 nm. **(G)** Phagocytosis assay showed that the 2-week-old RPE sheets, labeled by ZO-1 (green), were able to phagocytose FluoroSpheres (red). Scale bar = 20 μm.

Given the translational value of RPE induction, the differentiation and expansion capacity of the RPE component were further examined. We isolated the RPE spheres from retinal organoids at Week 5–7, when the RPE spheres could be clearly distinguished. The collected RPE spheres were then digested into small clusters and seeded. Consistent with previous reports (Hu et al., 2010; Liu et al., 2018), these cells proliferated vigorously and reached 100% confluence within 1 week, accompanied by pigment loss (Figure 8A). An increasing number of cells exhibited typical polygonal appearance in the following 2–4 weeks and the re-pigmentation gradually occurred in these cells with phase-bright borders, forming a honeycomb monolayer (Figure 8B). After that, spontaneously formed elevated domes were observed, suggesting the formation of apical-basal polarization and the underlying barrier function of RPE (Figure 8B’; Hu et al., 2010). Immunofluorescence analysis showed that the cells expressed naive RPE markers OTX2 and PAX6 in the first week during RPE expansion, but were negative for RPE65 (a mature RPE marker) (Figure 8C). The expression of RPE65 was detected when pigmentation re-appeared in cells, coupled with MITF and ZO-1 expression, indicating the maturation of RPE at this time (Figure 8D). Transmission electron microscopy showed that the RPE has developed microvilli and the abundant apical tight junctions (Figures 8E,F; Liu et al., 2018). Additionally, as shown in Figure 8G, through phagocytosis assay, we found that the RPE sheet was able to phagocytose polystyrene FluoSpheres, suggesting the functional ability of phagocytosis. In conclusion, mature and functional RPE could be yielded through the expansion of RPE spheres obtain from retinal organoids.

## Discussion

The SEAM system was reported as a two-dimensional eye-like colonies induction system without exogenous factors treatment. In the present study, our attempts showed that by improving the retinal components in the SEAM system, substantial 3D retinal organoids could be induced without the need of exogenous factors.

The differentiation of obtained retina oganoids mimics the early and late-phase of retinogenesis *in vivo*. From the morphologic changes observed under the microscope, two development stages with different growth rates could be defined. The early differentiation stage was characterized by a well-defined laminar neural epithelium and rapid growth rate, with ongoing thickness compression and relatively slower proliferation in the late differentiation stage. During retinogenesis *in vivo*, the differentiation capacity of neuroepithelial progenitors gradually changed; they tend to be restricted to the production of two or three types of cells at different stages, termed early- and late-phase retinogenesis (Livesey and Cepko, 2001; Bassett and Wallace, 2012). In our system, we found the development of RGCs, cone PRs, horizontal cells, and amacrine cells in the early differentiation stage; whereas in late stage, the occurrence of rod PRs, bipolar cells, and Müller glial cells were observed, resembling the early-born and late-born retinal lineages differentiation *in vivo*. Considering the great potential of retinal organoids in retinal replacement therapies, our study suggested that the underlying limited differentiation capacity should be taken into consideration when performing translational research using retinal organoids-derived retinal progenitor cells. To be noted that the efficiency of our protocol was based on hESC H9 cell line, the induction of other source of ESCs/iPSCs needs further test.

Along with retina organoid, our system enabled the spontaneous induction of RPE and ciliary margin-like domain. The interaction between the NR and the RPE is crucial for the ciliary margin formation, a potential retinal stem cells region in adult retina (Bhatia et al., 2010; Fischer et al., 2013). The Sasai group has previously developed an *in vitro* method for the co-induction of retinal organoids and the RPE that generates the CM. However, precise control of treatment time and drug dose are required, making it hard to replicate the results. We herein showed that the RPE could be co-induced in a recognizable domain, termed as zone 2, adjacent to the retinal organoid zone (zone 1) after modification based on SEAM method. By simply isolating zone 1 and zone 2, we could simultaneously induce retina organoids along with the RPE. Later, the interaction between the NR and the RPE domains resulted in the gradual formation of a CM-like domain at the NR-RPE boundary. Moreover, the obtained RPE spheres could be expanded and displayed similar marker expression, ultrastructural feature as native RPE, and exhibited the functional ability of phagocytosis. The presence of abundant apical tight junction and the elevated domes suggested that to some extent, our RPE sheets might be able to function as biological barrier. More biological characterization such as transepithelial resistance measurement is needed to verify the function of our RPE sheets. Taken together, we have established an exogenous-factors-free retinal organoid induction system, which also enabled the spontaneous generation of RPE and ciliary margin.

With the use of low-attachment microwell plates, we have improved the long-term survival rate, so as to improve the efficiency of obtaining retinal organoids with hair-like surface appendages, which indicated the developing PR outer segment. The gain of opsin expression, the patterned outer-segment formation, and the onset of phototransduction are considered to be hallmarks of the terminal differentiation of PRs (Swaroop et al., 2010). We herein have presented the morphological changes from precursors to the terminal differentiation of both PR types in our system, starting from the time when the outer-segment began to form. Moreover, unlike previous rod-cone analysis performed on slide sections, which may induce bias, we developed whole-mount staining and 3D reconstruction imaging to analyze the long-term maintained retina organoids. Combining the result of whole-mount staining and electron microscopy, it turned out that substantial mature PRs has developed, which contained mitochondria-rich inner segments-like, disk-containing rudimentary outer segments-like, and ribbon synapse structures. Additionally, the rod and cone PRs were unevenly distributed. To some extent, this may be related to the distance from NR-RPE hinge. We showed that rod PRs were mainly located in the hinge proximal region, while cone PRs occurred more frequently in the distal region; these were correlated with PR distribution *in vivo* (Szel et al., 2000).

Owing to the limitations of *in vitro* culture system, generating retinal organoid with microglia remains an unsolved problem (Cora et al., 2019; Zerti et al., 2020). In our induction system, we have found the expression of mesodermal marker Brachyury and classical microglia marker IBA1 in some cases (Supplementary Figure 3D), suggesting the presence of mesodermal progenitors and the transition status from mesodermal progenitors to microglia-like cells. However, they were located at the bottom of the NR spheres and no sign of migration into the inner retina was observed (Li et al., 2019). More attempts to verify the identity of these IBA1-expressing microglia is needed. In this regard, our integration of retinal generation with other ocular component may be a new solution for retinal organoid optimization.

Retinal organoid is a powerful system for the modeling of developmental mechanisms, for the testing and screening of drugs, and for the investigation of retinal replacement. Our exogenous-factors-free system features several advantages such as the fine reproducibility and repeatability, as well as the lower cost. Besides, when it comes to the application of our system, unpredictable cross reactivity with candidate signaling pathway, small molecule or drug could be minimum due to the absence of exogenous factor during retinal organoid induction.

In summary, we have established a simple and robust retinal organoid induction system based on the SEAM method. The overview of the cellular dynamics of the obtained retinal organoids presented above has demonstrated the potential to mimic early-phase and late-phase retinogenesis *in vivo*. In addition, our further modifications has enabled the generation of substantial mature PRs, the simultaneously induced RPE and the CM. Thus, we believe our exogenous-factors-free system may be a valuable new platform for studying retinogenesis and retinal translational application.

## Data Availability Statement

The original contributions presented in the study are included in the article/Supplementary Material, further inquiries can be directed to the corresponding author/s.

## Author Contributions

LL, SC, and YL contributed to conceptualization. JL and YC performed the induction experiments and writing. SO analyzed the data. JM performed the immunofluorescence staining imaging. HS maintained the induction system. All authors contributed to the article and approved the submitted version.

## Conflict of Interest

The authors declare that the research was conducted in the absence of any commercial or financial relationships that could be construed as a potential conflict of interest.

## Publisher’s Note

All claims expressed in this article are solely those of the authors and do not necessarily represent those of their affiliated organizations, or those of the publisher, the editors and the reviewers. Any product that may be evaluated in this article, or claim that may be made by its manufacturer, is not guaranteed or endorsed by the publisher.
