# Supplementary material for: Generation and Staging of Human Retinal Organoids Based on Self-Formed Ectodermal Autonomous Multi-Zone System
Source: Front Cell Dev Biol. 2021 Sep 22;9:732382. doi: 10.3389/fcell.2021.732382 (PMC8493070; doi:10.3389/fcell.2021.732382)
Supplement: Supplementary file 1 [file Image_1.pdf]

Generation and Staging of Human Retinal Organoids Based on Self-formed Ectodermal Autonomous Multi-zone (SEAM) System

Supplementary Material

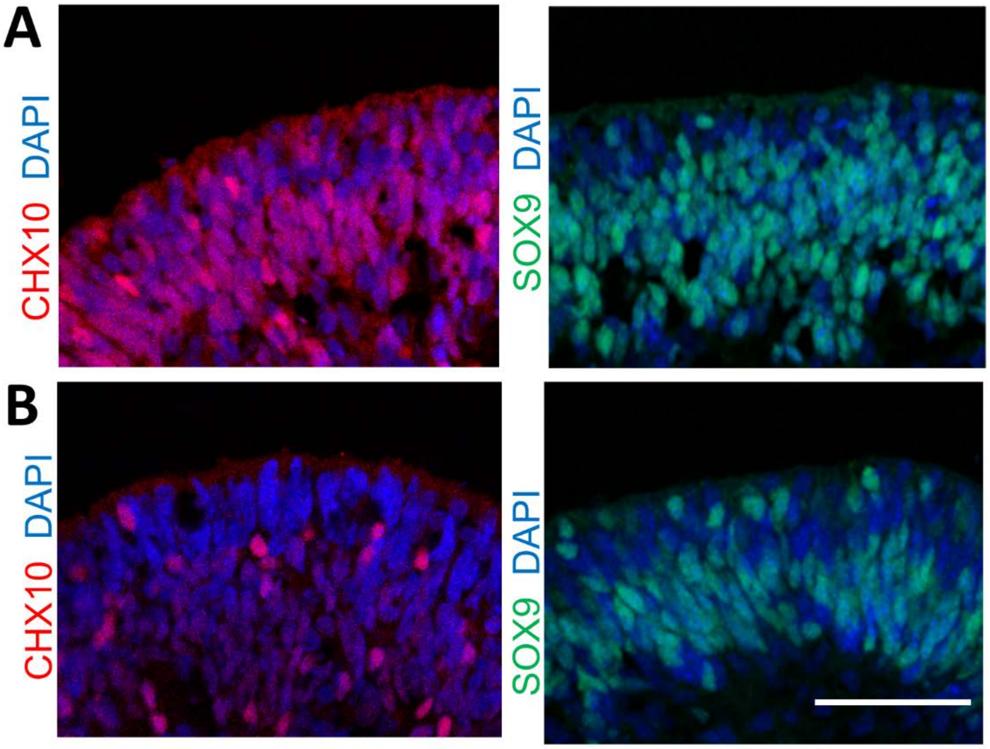

Supplementary Figure 1. Expression dynamics of neural progenitor cell markers CHX10 and SOX9. (A) CHX10 (red) and SOX9 (green) at Week 5 labeled the retinal progenitor cells, which were found throughout the neuroepithelium. (B) CHX10- (red) and SOX9- (green) labeled cells tended to be restricted to the inner side of neuroepithelium at Week 7. Scale bar = 50 μm.

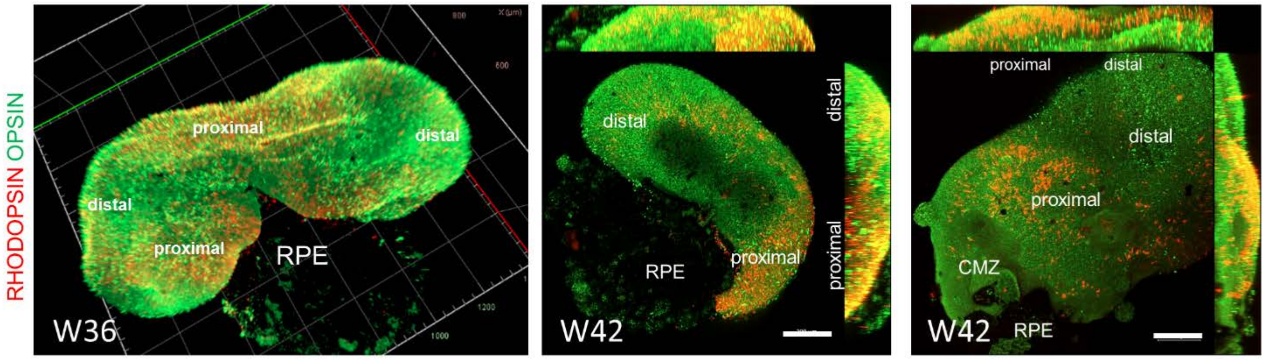

Supplementary Figure 2. More images of whole mount staining of Opsin (green) and Rhodopsin (red) showed a substantial number of mature cone and rod photoreceptor cells at Week 36, Week 42 and Week 42, respectively. Scale bar = 200 µm.

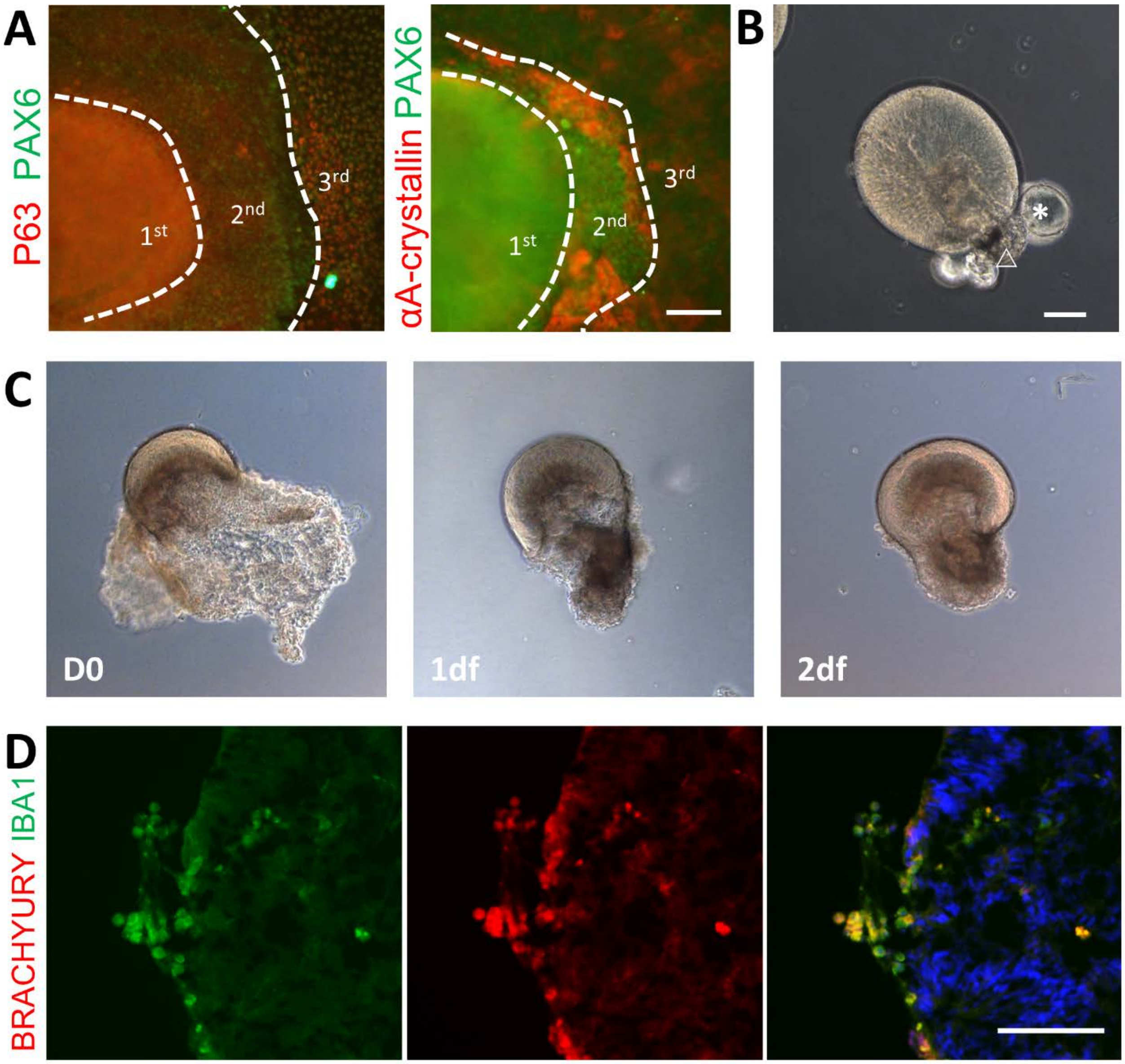

Supplementary Figure 3. Autonomous generation of multiple-ocular cells. (A) Zone 3 is marked by co-staining with PAX6 (green) and P63 (red), indicating the onset of corneal precursors. At the boundary of zone 2 and zone 3, a group of cells co-stained with PAX6 (green) and P63 (red) is shown, suggesting the derivation of lens primordial cells on Day 28. (B) Representative images showing that zone 1–3 isolation could yield retinal organoids with RPE spheres (triangles) and lens/cornea spheres (asterisks). (C) Phase-contrast images showing that during the first 3 days after zone 1–3 suspension, the accessory aggregates gradually altered into spheres. (D) Immunofluorescent staining of the expression of mesodermal marker Brachyury and classical microglia marker IBA1 indicated the presence of mesodermal progenitors and the transition status from mesodermal progenitors to microglia-like cells on Day 48. Scale bar = 50 μm.
