# Supplementary material for: Generation and Staging of Human Retinal Organoids Based on Self-Formed Ectodermal Autonomous Multi-Zone System
Source: Front Cell Dev Biol. 2021 Sep 22;9:732382. doi: 10.3389/fcell.2021.732382 (PMC8493070; doi:10.3389/fcell.2021.732382)
Supplement: Supplementary file 2 [file Table_1.PDF]

Supplementary Table 1. The primary antibodies are listed.

Antibody	source	catalog number	dilution
SIX3	Santa Cruz	sc398797	1:200
PAX6	Covance	PRB-278P	1:1000
CHX10	Millipore	AB9014	1:100
OTX2	Santa Cruz	sc30659	1:100
SOX2	Santa Cruz	sc17320	1:200
MITF	Thermo Fisher Scientific	MS-771-P1	1:100
Ki67	Thermo Fisher Scientific	18-0191Z	1:100
BRN3	Santa Cruz	SC-6026	1:1000
Tubulin β3 (Tuj1)	BioLegend	801201	1:1000
CRX	Abcam	ab78662	1:100
AP-2α	Santa Cruz	sc12726	1:200
PROX1	R&D Systems	AF2727	1:100
Calbindin	Abcam	ab11426	1:100
Recoverin	Millipore	AB5585	1:1000
SOX9	Millipore	AB5535	1:1000
Opsin Green/Red (Opsin G/R)	Abcam	ab5405	1:100
Rhodopsin	Sigma	MABN15	1:1000
Arrestin-C	Santa Cruz	sc293296	1:100
VGLUT1	Synaptic Systems	135302	1:500
CX43	Santa Cruz	sc271837	1:500
RPE65	Abcam	ab13826	1:100
ZO-1	Cell Signaling Technology	cst13663	1:400
P63	Abcam	ab735	1:100
αA-crystalline	Santa Cruz	sc28306	1:200
Brachyury	Santa Cruz	sc166962	1:100
IBA-1	Wako Chemicals	019-19741	1:200
